# Spider-Venom Peptides as Bioinsecticides

**DOI:** 10.3390/toxins4030191

**Published:** 2012-03-22

**Authors:** Monique J. Windley, Volker Herzig, Sławomir A. Dziemborowicz, Margaret C. Hardy, Glenn F. King, Graham M. Nicholson

**Affiliations:** 1 Neurotoxin Research Group, School of Medical & Molecular Biosciences, University of Technology, Sydney, Broadway NSW 2007, Australia; Email: monique.windley@student.uts.edu.au (M.J.W.); slawomir.dziemborowicz@student.uts.edu.au (S.A.D.); 2 Institute for Molecular Bioscience, The University of Queensland, St Lucia, Queensland, 4072, Australia; Email: v.herzig@imb.uq.edu.au (V.H.); m.hardy@uq.edu.au (M.C.H.)

**Keywords:** spider venom, peptide, insecticidal, bioinsecticides, cystine knot, pest control

## Abstract

Over 10,000 arthropod species are currently considered to be pest organisms. They are estimated to contribute to the destruction of ~14% of the world’s annual crop production and transmit many pathogens. Presently, arthropod pests of agricultural and health significance are controlled predominantly through the use of chemical insecticides. Unfortunately, the widespread use of these agrochemicals has resulted in genetic selection pressure that has led to the development of insecticide-resistant arthropods, as well as concerns over human health and the environment. Bioinsecticides represent a new generation of insecticides that utilise organisms or their derivatives (e.g., transgenic plants, recombinant baculoviruses, toxin-fusion proteins and peptidomimetics) and show promise as environmentally-friendly alternatives to conventional agrochemicals. Spider-venom peptides are now being investigated as potential sources of bioinsecticides. With an estimated 100,000 species, spiders are one of the most successful arthropod predators. Their venom has proven to be a rich source of hyperstable insecticidal mini-proteins that cause insect paralysis or lethality through the modulation of ion channels, receptors and enzymes. Many newly characterized insecticidal spider toxins target novel sites in insects. Here we review the structure and pharmacology of these toxins and discuss the potential of this vast peptide library for the discovery of novel bioinsecticides.

## 1. The Global Insect Pest Problem

### 1.1. Agricultural Pests

Arthropods are the most successful and diverse group of animals, with an estimated 2.8–10 million global species [1]. While only around 10,000 species are recognised as crop pests, approximately 14% of global crop loss and 20% of damage to stored food grains are due to insects [2,3]. This results in an estimated USD100 billion in damage each year [4]. Phytophagous (plant-eating) arthropods are the major cause of this crop loss. These include insect species from the Orders Coleoptera (beetles), Orthoptera (locusts and grasshoppers) and Lepidoptera (moths and butterflies) [5]. While the larval forms of lepidopterans are considered the most destructive [6], with 40% of chemical insecticides directed against heliothines [7], insect species from the Order Diptera (true flies), Hemiptera (true bugs), Thysanoptera (thrips) and Acarina (mites) are also recognised as important crop pests [6,8]. The crop loss caused by insect pest damage diminishes our ability to meet the ever-increasing demand for food production to sustain the world’s population, which is expected to grow from ~7 billion to around 9.31 billion people in the next 40 years (U.N. Department of Economic and Social Affairs; http://esa.un.org/unpd/wpp/unpp/panel_population.htm).

### 1.2. Vectors of Disease

A number of arthropod pests act as disease vectors for the transmission of infectious diseases of human and veterinary health importance [9]. In particular, insects belonging to the Order Diptera, such as mosquitoes, midges and flies, are major disease vectors [10,11,12]. Hematophagous (blood-sucking) dipterans are collectively responsible for a wide variety of infections known to cause human morbidity and mortality, including malaria, dengue fever, West Nile virus, yellow fever, filariasis, leishmaniasis, Japanese encephalitis and African trypanosomiasis [10,11,12]. Other disease vectors include ticks, fleas, lice and triatomid bugs [13], which are responsible for the transmission of infectious diseases such as Lyme disease, ehrlichiosis, various rickettsioses, Rocky mountain spotted fever, tularemia, bubonic plague, Chagas disease and Bartonella [10,11,14,15,16,17]. Newly emerging diseases, such as onchocerciasis, Barmah Forest virus, Japanese spotted fever and dengue-dengue hemorrhagic fever are also vectored by arthropods [10]. Of these infectious diseases, malaria best exemplifies the need for insect pest control due to the fact that 3.3 billion people—almost 50% of the world population—live in areas at risk of transmission [18]. There were 216 million cases of malaria in 2010, averaging one death every minute, most of whom were children under the age of 5 [19]. Treatment is available, yet the infection still accounts for 20% of all childhood deaths in Africa [20]. 

Arthropod-mediated viral, rickettsial, bacterial and protozoan diseases pose not only a threat to human health, but also have consequences for global food production. Poultry and livestock diseases such as African swine fever, Akabane disease, bovine ephemeral fever, equine encephalitis, blue tongue fever and epizootic hemorrhagic fever all have the potential to compromise animal health. Symptoms range from lameness, blindness, wasting, congenital defects, spontaneous abortion and sterility to death, with infected livestock often being destroyed [21]. Currently, arthropod pest control and eradication programs rely on synthetic chemical insecticides as a means of reducing, if not eliminating, the prevalence of these debilitating diseases in humans and animals.

## 2. Agrochemical Insecticides: Current Challenges to Insect Pest Control

Chemical insecticides were first introduced in the 1940s and they remain the major method for controlling insect pests. Chemical insecticides were seen as promising tools for insect control with the remarkable success of DDT in malaria eradication programs [22,23]. Organophosphates were then introduced in the 1960s [23]. The widespread use of organophosphates and other chemical insecticides in agriculture and malaria eradication programs provided a quick and relatively cheap solution to the growing insect pest problem. However, major problems with the use of agrochemicals have arisen including (i) a lack of phyletic specificity resulting in human health and environmental impacts, and (ii) a lack of diversity in the bioactivity of these compounds leading to insecticide resistance. 

### 2.1. Health Consequences and Environmental Impacts

To provide effective and safe insect control it is important that insecticides act with high affinity only at specific sites within the target invertebrate. Unfortunately, the majority of current agrochemicals act on targets conserved across insects and non-target organisms, including humans. As a consequence, acute toxicity is well documented in both animal models and humans. For example, in the developing world, over 250,000 people die each year from suicide and deliberate self-harm using insecticides and other pesticides. [24,25]. These deaths are responsible for about a third of suicides globally [24] and the World Health Organization (WHO) now recognizes pesticide poisoning to be the single most important means of suicide worldwide [26].

The effects of chronic exposure to residual chemical pesticides, however, remain controversial [27]. Epidemiological studies have purported to show a link between exposure to chemical pesticides and the development of cancers including pancreatic cancer, multiple myeloma, leukaemia, ovarian cancer and prostate cancer (for a review see reference [28]). However, the evidence is not substantial and currently only arsenic-containing insecticides are considered carcinogenic, while others are only suspected of carcinogenicity [29]. There are also possible links between chronic pesticide exposure and congenital defects [28,30], preterm birth [31], Parkinson’s disease [32,33,34,35,36], and neuropsychological dysfunctions (for a review see reference [37]). 

Adverse environmental effects are also of concern. Due to the indiscriminate actions of some agrochemical insecticides, beneficial insects (e.g., pollinators such as bees and butterflies), birds, aquatic invertebrates and fish can also succumb to the toxic effects of these agents either through direct, or indirect, exposure in the form of spray drift, run-off or leaching [38,39,40]. Some agrochemicals also persist in the environment, with insecticides such as DDT highlighting the deleterious effects of bioaccumulation. These environmental problems along with human health concerns have seen the de-registration or use-cancellation of 169 insecticides between January 2005 and December 2009, with only 9 new insecticides registered during the same period [41]. 

### 2.2. Insecticide Resistance

The vast majority of agrochemicals act on a single target within the insect nervous system. Indeed chemical insecticides interact with just one of five main targets—voltage gated sodium (Na_V_) channels, glutamate receptors, γ-aminobutyric acid (GABA) receptors, nicotinic acetylcholine receptors and acetylcholinesterases [42]—although a new class of insecticide has recently been developed that targets ryanodine receptors [43]. As a result, the use of agrochemicals with so few targets has promoted the evolution of resistance to a number of insecticide families [17,44]. There are multiple ways that this insecticide resistance can arise: (i) increased metabolic detoxification, (ii) decreased target sensitivity, and/or (iii) increased sequestration or lowered insecticide bioavailability [17,44]. The molecular mechanisms responsible for these increases in resistance include point mutations in the ion channel of the GABA receptor or Na_V_ channel, mutations in the active site of acetylcholinesterase, amplification of esterase genes, and mutations causing up-regulation of detoxification enzymes [17,44,45,46]. Unfortunately, resistance has now arisen in almost all insect vector species [47,48]. In particular, increases in the number of surviving insect vectors following treatment with insecticides is predicted to directly influence the resurgence [49], or challenge the management, of vector-borne diseases [47].

These problems indicate the need to identify new and safe insecticidal lead compounds, validate novel insecticidal targets and develop alternate methods of effective insect control. Therefore, it is crucial that we identify novel insecticides that can exploit subtle differences in targets that are conserved between insects and vertebrates, or agents that target structures only found in insects.

## 3. Bioinsecticides as Natural Insect Pest Control Agents

Bioinsecticides are being investigated as potentially more efficacious and safer alternatives to chemical insecticides. Bioinsecticides are natural organisms, or their metabolic products, that can be employed for the control of insect pests. One aim is to develop bioinsecticides to help mitigate environmental concerns associated with persistent, broad-spectrum chemical insecticides and provide new control options for insecticide-resistant pest insects [50]. Furthermore, bioinsecticides have the potential to improve the efficacy of current pest management programs, and in some cases exhibit synergism with existing integrated pest management (IPM) techniques [51]. 

In 2009 the global pesticide market was valued at approximately USD43 billion, with a predicted compound annual growth rate (CAGR) of 3.6% and a projected value of USD51 billion in 2014 [52]. In contrast, the global biopesticide sector has grown more strongly, with a CAGR of 15.6% and an increase in value from USD1.6 billion in 2009 to an estimated USD3.3 billion in 2014. In 2006, orchard crops had the largest share of biopesticide use at 55%, and in the same year biopesticides represented roughly 2.5% of the global pesticide market [53]. However, synthetic pesticides still retain the highest market share, with a CAGR of 3% leading to an estimated value of USD48 billion in 2014 [52]. Nevertheless, the 5-fold higher CAGR for biopesticides has resulted in increased interest in this sector of the market [53]. 

The potential sources of biopesticides include microbes (viral, fungal, bacterial), entomophagous nematodes, plant-derived products, insect pheromones and insect resistance genes expressed in crops (for a review see reference [54]). In particular, insecticidal toxins derived from insect predators and parasitoids are of growing interest in the development of bioinsecticides, and these include peptide neurotoxins derived from the venoms of scorpions [55], parasitic wasps [56], the straw itch mite [57], and spiders [58,59]. Currently, there is a great deal of interest in spider venoms as they comprise an extensive library of potent insecticidal, neurotoxic peptides.

## 4. Spider Venoms: Sources of Novel Bioinsecticides

**Figure 1:**
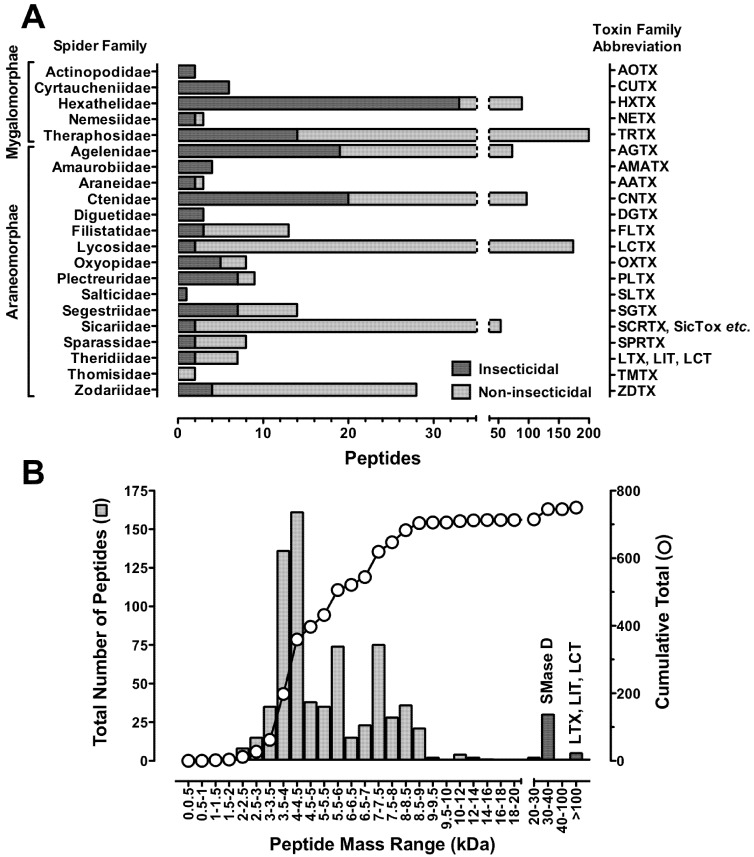
Distribution of spider-venom peptides. (**A**) Distribution of characterized araneomorph and mygalomorph spider-venom peptides organized by spider family. Abbreviated toxin family names are indicated on the right hand ordinate. Bars in dark grey indicate insecticidal toxins, while light grey bars are non-insecticidal peptides, including those with unknown activity, from the same spider family; (**B**) Mass distribution of characterized spider-venom peptides. Masses represent the monoisotopic oxidized mass sorted into 500 Da bins. The overlaid curve shows the cumulative total number of peptides. Dark grey columns show mass ranges dominated by proteins with sphingomyelinase D (SMase D) activity from *Loxosceles* spp., as well as latrotoxins (LTX), latroinsectoxins (LIT) and latrocrustatoxins (LCT) from *Latrodectus* spp. *N-*terminal fragments are not included in the data. Note the discontinuous abscissa in both panels. Data were collated from the ArachnoServer 2.0 Spider Toxin Database (www.arachnoserver.org; [70], accessed on 20 January 2012).

Spiders are ancient creatures that evolved from an arachnid ancestor around 300 million years ago during the Carboniferous period. This highlights the long evolutionary timescale over which spiders have evolved their complex venom. Spiders are the most speciose venomous animal and along with predatory beetles are the most successful terrestrial predators, with over 42,000 extant species described to date [60]. This may be an under-representation of their true speciation, with about four times as many species predicted to exist, but not yet characterised [61]. One of the major features contributing to the overall success of spiders is the production of a highly toxic venom from their venom glands that they employ to subdue prey and deter predators. Since they rely completely on predation as a trophic strategy, spiders have evolved a complex pre-optimized combinatorial library of enzymes, neurotoxins and cytolytic compounds in their venom glands [62,63,64,65,66,67,68,69]. These venom components fall into three classes delineated by their molecular mass: (i) low molecular mass acylpolyamines and other nonpeptidic molecules (<1 kDa), (ii) disulfide-rich neurotoxins and linear cytolytic peptides (1–10 kDa), and (iii) high molecular mass proteins (>30 kDa) comprising mainly enzymes and neurotoxins. Most spider venoms are dominated by small disulfide-rich peptide neurotoxins (Figure 1B), and these are the largest and most extensively studied group of spider toxins. 

To date, around 800 peptide toxins from 78 spider species have been described in ArachnoServer 2.0 (www.arachnoserver.org), a curated database containing available information on spider-venom peptides and proteins [70,71]. These toxins were isolated from the venom of 20 of the 110 extant spider families, including representatives from the two major infraorders Araneomorphae (“modern” spiders) and Mygalomorphae (“primitive” spiders) (Figure 1A). Araneomorphs represent >90% of all known spider species. However, mygalomorphs are a more sustainable and convenient source of venom due to their large venom glands and their longevity (they can live for over 25 years). In recent years, it has become clear that spider venoms are considerably more complex than previously appreciated, with some venoms containing more than 1000 distinct peptides [72]. If one uses a conservative estimate of 100,000 species and 200 peptides per venom, then spider venoms may contain upwards of 10 million bioactive peptides [73]. Less than 0.01% of this proteomic diversity has been explored to date.

Spiders utilize their venoms to paralyze and/or kill prey or predators as rapidly as possible. Therefore their venoms are particularly rich in neurotoxins that rapidly modify ion conductance (ion channel toxins), and to a lesser extent affect neurotransmitter exocytosis (presynaptic toxins). However, like scorpion toxins, they appear to lack significant numbers of postsynaptic toxins that block the action of neurotransmitters, which are particularly common in the venom of snakes and, to a lesser extent, marine cone snails. Many of these spider peptide toxins are selectively insecticidal. In particular, insect-selective toxins have been patented for their possible use as bioinsecticidal agents for the control of phytophagous pests or insect vectors [74]. The focus of this review is the discovery, processing, structure, and function of insecticidal spider-venom peptides. In particular, it will detail the site and mechanism of their action, the molecular determinants for their pharmacology, and discuss the application of these peptides in the development of novel bioinsecticides. 

## 5. Spider-Venom Peptide Nomenclature

Recently there has been an exponential increase in the number of spider toxins that have been reported in the literature [75]. This has resulted from the advent of modern high-throughput analytical techniques involving proteomic, transcriptomic and genomic approaches. As a result, a rational nomenclature system based on a Greek letter “activity prefix’ together with a toxin name based on the family, genus and species of the spider has been recently proposed [75]. This nomenclature has been adopted by UniProtKB and ArachnoServer 2.0. This review will employ this new nomenclature to facilitate identification of orthologs and paralogs, but we will also provide original names of the toxin. In addition, readers are directed to relevant entries in the ArachnoServer 2.0 database for original literature references, biological activity, molecular targets, sequence and 3D structure (where known).

## 6. Structure of the Precursor Spider-Venom Peptide and Post-Translational Processing

**Figure 2:**
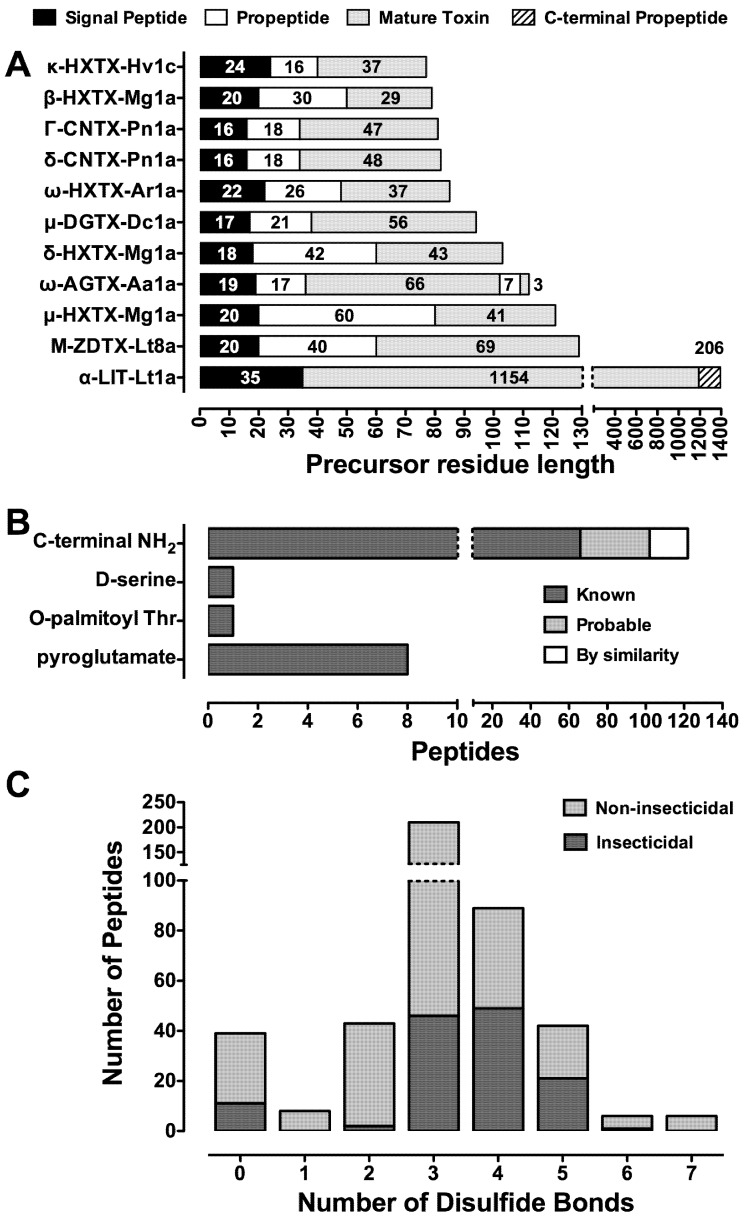
Precursor architecture and posttranslational modifications in spider-venom peptides. (**A**) All insecticidal spider-venom toxins display a classical prepropeptide paradigm except LITs (e.g., α-LIT-Lt1a) from *Latrodectus* spp. ω-AGTX-Aa1a from *Agelenopsis aperta* is a heterodimer consisting of a 66-residue major chain that is linked via a disulfide bond to a 3-residue minor chain; (**B**) Known PTMs in spider-venom peptides (dark grey bars) as well as probable PTMs (light grey bars) and those predicted from sequence homology (white bars); (**C**) Distribution of the number of disulfide bonds found in insecticidal spider toxins (dark grey bars) and non-insecticidal peptides (light grey bars). Peptides with unknown disulfide connectivity are not shown. Note the discontinuous axes in all panels. Data were collated from the ArachnoServer 2.0 Spider Toxin Database (www.arachnoserver.org; [70], accessed on 20 January 2012).

Similar to peptides from marine cone snails and sea anemones, spider-venom peptides are translated as precursors that undergo post-translational modification to yield the mature toxin [68]. These precursors are typically composed of an *N-*terminal signal peptide of 15–47 residues that generally precedes a propeptide region rich in acidic residues and of highly variable length, followed by a single downstream copy of the mature toxin sequence (Figure 2A). However, some larger spider-venom proteins do not contain a propeptide region in their precursor.

It appears that during evolution toxin diversity is maintained through gene duplication followed by focal hypermutation in the mature peptide region while conserving the basic disulfide framework [68]. Hypermutation of the mature toxin sequence often gives rise to new pharmacological activity. Peptide libraries of toxin paralogs are maintained, with spider species capable of expressing up to 26 variants (homologs/isoforms) of a single peptide toxin (e.g., U_2_-AGTX-Ao1a to -Ao1z [70]). Despite this high diversity, the signal sequence within the prepropeptide and the Cys residues responsible for correct protein folding are highly conserved [68]. The signal peptide is presumably conserved since its role is to direct the precursor to a specific secretory pathway to ensure correct peptide folding. The specific role(s) of the propeptide region is still not understood but it may enhance folding of the mature toxin and provide signals for post-translational modifications (PTMs) such as *N-*terminal pyroglutamate formation, palmitoylation, and *C-*terminal trimming and amidation (Figure 2B). However, insecticidal spider peptide toxins have only been observed with palmitoylation and *C-*terminal trimming/amidation. In the case of the high molecular mass latroinsectoxins, from *Latrodectus* spp. (widow spiders), the *N*-terminal propeptide is absent (Figure 2A). These mechanisms have allowed spiders to evolve vast libraries of peptides with variable pharmacological activity. 

## 7. Structural Motifs of Spider-Venom Peptides: Variations on an Ancestral Fold

**Figure 3:**
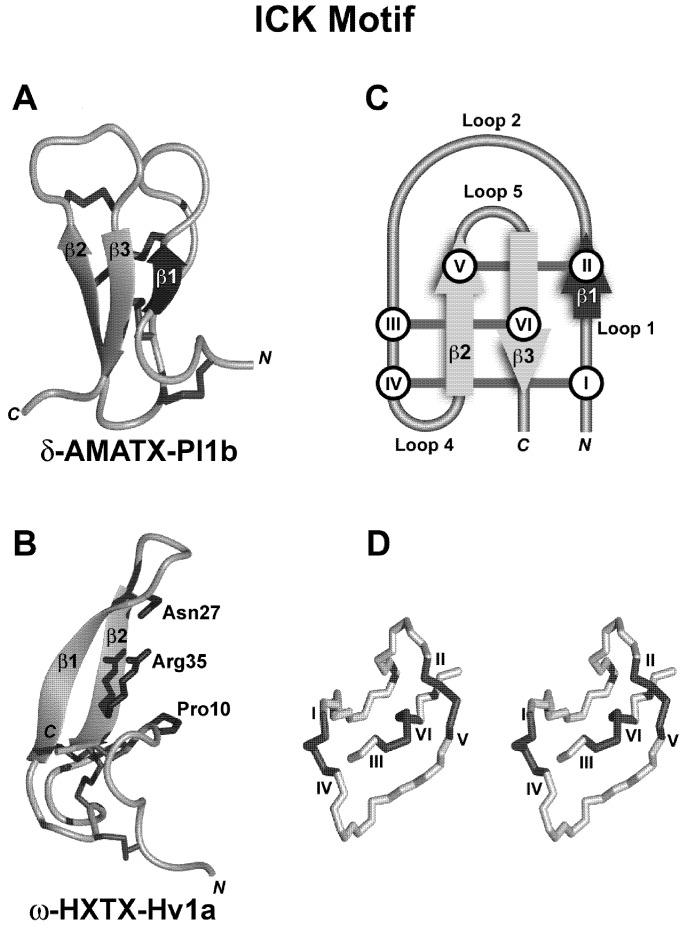
The ICK structural motif. Left-hand panels (**A**,**B**) show a schematic view of the 3D structures of typical representatives of the ICK structural motif: (**A**) The insecticidal peptide δ-AMATX-Pl1b (PDB 1V91) and (**B**) the insecticidal peptide ω-HXTX-Hv1a (formerly ω-ACTX-Hv1a; PDB 1AXH) showing the major pharmacophore residues. Panel (**C)** shows a schematic representation of the ICK motif depicting the formation of the cystine-knot and possible addition of the third β-strand. The dark arrow (β1) represents the additional β-strand not always present in ICK spider-venom peptides (*i.e.*, present in **A** but not **B**). (**D**) Stereoview of the cystine-knot motif of κ-TRTX-Scg1a (formerly SGTx1). In all panels, β-strands are shown as gray arrows and disulfide bridges connecting cysteine residues are shown as dark gray lines with roman numerals.

Around 90% of spider-venom toxins are compact globular proteins possessing several disulfide bridges. The number of disulfide bridges ranges from one to seven, but nearly 60% of all toxins have three bridges (Figure 2C). These peptides, predominantly targeting voltage-activated ion channels, often contain a “disulfide pseudo-knot” which places them in a class of toxins and inhibitory polypeptides with an “inhibitor cystine-knot” (ICK) motif [76]. This structural motif is normally exemplified by a triple-stranded, antiparallel β-sheet stabilized by disulfide bridges. Since not all ICK peptides exhibit the *N-*terminal β-strand (β1 in Figure 3Cb), a modified definition composed of “an antiparallel β-hairpin stabilized by a cystine-knot” without a mandatory third β-sheet has been proposed [76,77,78]. The three disulfide bridges and intervening backbone form a pseudo-knot consisting of a ring (Cys_I_-Cys_IV_, Cys_II_-Cys_V_) penetrated by a third disulfide bridge (Cys_III_-Cys_VI_) [76]; see Figure 3Db. The ICK has a consensus sequence of -Cys-X_3–7_-Cys-X_3–8_-Cys-X_0–7_-Cys-X_1–4_-Cys-X_4–13_-Cys- where X is any amino acid [76]. Within this fold-class, the biological activities of spider ICK toxins are quite diverse with activity at voltage-activated sodium (Na_V_), calcium (Ca_V_), and potassium (K_V_) channels, acid-sensing ion channels (ASICs), transient receptor potential (TRP) channels, and mechanosensitive channels (MSCs) (see below). This highlights the observation that different biological functions are often grafted onto the same, or similar, structural scaffolds.

Another structural fold has been defined for spider toxins. The disulfide-directed β-hairpin (DDH) fold lacks the disulfide knot and is composed of a double-stranded antiparallel β-hairpin stabilized by two mandatory disulfide bridges with a current consensus sequence of -CysX_4–19_-CysX_2_(G or P)X_2_-CysX_4–19_-Cys-, where X is any amino acid (Figure 3A,B). The ICK motif appears to have evolved from this simpler canonical ancestral fold [79,80]. This DDH fold has been observed in a range of peptides with unknown targets such as the MIT-like U_1_-HXTX-Hv1a [81] and U_1_-TRTX-Lp1a and -Lp1b [63], and the insecticidal toxins U_1_-TRTX-Hh1 toxins, U_1_-TRTX-Asp1f and -Asp1g [63]. The ICK fold in particular creates hyperstable mini-proteins that are typically resistant to extremes of pH, organic solvents, and high temperatures [82]. However, from a bioinsecticide perspective, their most important property is their resistance to proteases. Specific differences in the DDH and ICK structural folds, determined by the spacing between cysteine residues and their connectivity, is critical for the presentation of key functional residues to the target. This together with their protease resistance and compact nature provides an effective scaffold for the design of bioinsecticides, including peptidomimetics, as well as molecular tools and therapeutics [76]. Finally, another structural motif that has been reported in spider venoms is the Kunitz-type toxin motif characterised by an *N-*terminal 3_10_ helix, *C-*terminal α-helix and a triple-stranded antiparallel β-sheet with a C_I_-C_VI_, C_II_-C_IV_, C_III_-C_V_ disulfide bonding pattern. Peptides and proteins with this motif exhibit potassium channel blocking activity and also act as serine protease inhibitors. This structural fold has been discovered in toxins from a variety of other venomous animals including cone snails, scorpions, sea anemones, snakes, ticks, and wasps, and but has so far only been reported in the venoms of two theraphosid spiders (*Haplopelma schmidti* and *H. hainanum*) and one araneid spider (*Araneus ventricosus*) [83].

## 8. Insecticidal Targets of Spider Neurotoxins

**Figure 4:**
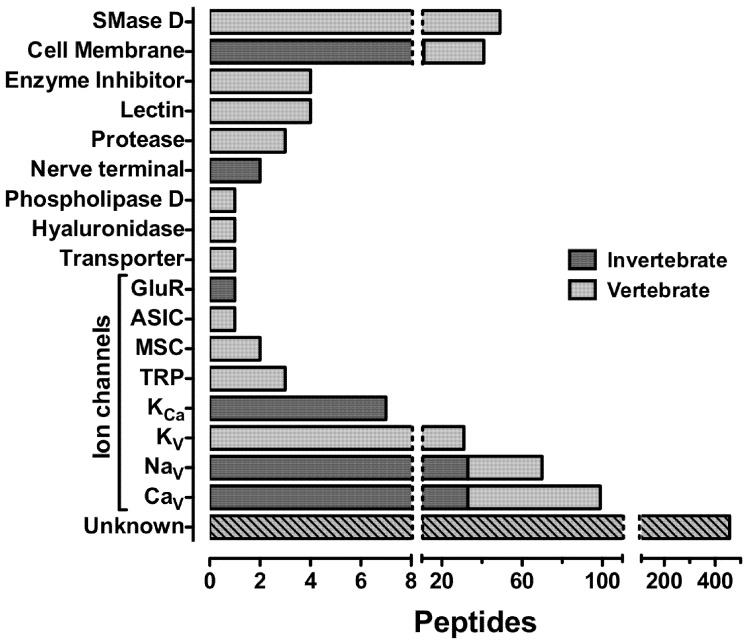
Molecular targets of spider toxins. Dark grey bars represent invertebrate targets of insecticidal spider-venom toxins, while light grey bars represent vertebrate targets. Toxins may have more than one target and may be phylum-selective or non-selective. Abbreviations: SMase D, sphingomyelinase D; GluR, glutamate receptor; ASIC, acid-sensing ion channel; MSC, mechanosensitive channel; TRP, transient receptor potential; K_Ca_, calcium-activated potassium channel. Note the discontinuous abscissa. Data were collated from the ArachnoServer 2.0 Spider Toxin Database (www.arachnoserver.org; [70], accessed on 19 January 2012).

There are predicted to be at least 10 million bioactive spider-venom peptides [73] of which only 800 have been characterized. Of the 800 peptides in the ArachnoServer 2.0 Database, 136 are insecticidal with 38 being insect-selective, 34 non-selective and 64 of unknown phyletic selectivity (these data do not include homologs whose activity and phyletic selectivity is yet to be determined). Of the insecticidal spider toxins the molecular target has only been identified for 85 (63%). To date, the most common identified targets of insecticidal spider-venom toxins are Na_V_ channels (*n* = 33), Ca_V_ channels (*n* = 33), the lipid bilayer (*n* = 11), calcium-activated potassium (K_Ca_) channels (*n* = 7), presynaptic nerve terminals (*n* = 2) and *N-*methyl-D-aspartate (NMDA) receptors (*n* = 1); see Figure 4. However, these statistics may be skewed by the rather limited range of targets that have been assayed to date. With advances in venom screening technologies [84], it is likely that spider toxins with novel molecular targets will be discovered in the near future. In the subsequent sections we review the structure and pharmacology of some of the insect-selective toxins that have been identified in spider venoms.

### 8.1. Spider-Venom Peptides Targeting Insect NaV Channels

Mammalian and insect Na_V_ channels mediate inward sodium conductance during the depolarisation phase of the action potential and regulate a wide range of physiological processes [85,86]. The crystal structure of a bacterial Na_V_ channel has recently been determined and it has confirmed the structural basis for voltage-dependent gating, ion selectivity and drug block of the channel [87]. The Na_V_ channel contains a pore forming α-subunit, associated with one or two auxiliary β-subunits [88]. The α-subunit has four homologous domains (I–IV) that are further divided into six transmembrane sections (S1–S6). The voltage-sensing domain is composed of the S1–S4 segments that flank the pore module comprising the S5 and S6 segments. The re-entrant P-loop between S5 and S6 forms the narrow ion-selectivity filter at the extracellular end of the pore [88]. The S4 segment acts as the voltage sensor by locating charged amino acids within the membrane electric field that undergo outward displacement in response to depolarization and initiate opening of the ion pore [89]. Sodium channel inactivation is mediated by a short intracellular loop connecting domains III and IV [90,91].

To date, nine mammalian Na_V_ channels (Na_V_1.1–1.9) have been cloned and, in all cases except Na_V_1.9, functionally expressed [92]. Consequently the structural, functional and pharmacological diversity of mammalian Na_V_ channels is achieved primarily through expression of multiple genes. In contrast, insects appear to rely upon extensive alternative splicing and RNA editing of a single para Na_V_ channel gene to provide channels with different functional properties. For example, gene splicing of the Na_V_ channels has been observed at nine different sites in *Drosophila* [93,94]. This has the potential of leading to 100 distinct variants of the insect Na_V_ channel.

The wide range of para Na_V_ channels are highly conserved across various insect orders, with sequence identities of 87–98% [95]. Hence, insecticides targeting insect Na_V_ channels have broad toxicity across diverse insect orders. In contrast, para Na_V_ channels have only low levels of sequence identity (50–60%) with mammalian Na_V_1.1–1.9 channels [95]. As a result, insect and mammalian Na_V_ channels are distinguishable pharmacologically by the selective action of several chemical insecticides. These include DDT, pyrethroids, *N-*alkylamides, oxadiazines and dihydropyazoles [42,96,97], as well as a growing range of insect-selective Na_V_ channel toxins including those derived from spider venoms. Much of the structure and function of Na_V_ channels have also been determined using toxins derived from a range of animal venoms and plants. These molecular probes have enabled identification of at least seven allosterically coupled neurotoxin binding sites, referred to as neurotoxin receptor sites 1–7, of which three sites bind spider-venom peptides [98,99,100]. Toxins targeting Na_V_ channels are expressed in most families of araneomorph and mygalomorph spiders, suggesting an early development during venom evolution. These spider toxins modulate neuronal excitability, resulting in paralysis (both flaccid and excitatory) and death in insects. Importantly, some spider toxins are highly selective for these three neurotoxin receptor sites on insect Na_V_ channels and thus insect-selective spider neurotoxins have potential to be developed as bioinsecticides. Importantly, the three neurotoxin receptor sites do not correspond to the site targeted by pyrethroids, DDT or DDT analogues (site 7) [98] so the possibility of cross resistance between spider toxins and pyrethroids/DDT is negligible.

#### 8.1.1. Spider-Venom Peptides Targeting Insect NaV Channel Site-1: Pore Blockers

Site-1 neurotoxins, like the guanidinium-containing alkaloid neurotoxin tetrodotoxin (TTX), physically occlude the pore region of the channel and are referred to as pore blockers. μ-Theraphotoxin-Hhn2b (µ-TRTX-Hhn2b; formerly hainantoxin-I) is the most abundant component within the crude venom of the Chinese black earth tiger tarantula *Haplopelma hainanum* [101] and blocks insect channels with high affinity [102]. It displays 15-fold selectivity for the *Drosophila* para (DmNa_V_1) channel compared with rat Na_V_1.2, with no effect on rat Na_V_1.1 and Na_V_1.4–1.8 channels [102]. It does not appear to alter ion selectivity, nor alter the voltage-dependence of activation or inactivation kinetics. However, µ-TRTX-Hhn2b is associated with a hyperpolarizing shift in the voltage dependence of steady-state Na_V_ channel inactivation that stabilizes the channel in the inactivated (closed) state and inhibits Na^+^ conductance [102]. It has been claimed that µ-TRTX-Hhn2b is the first spider toxin to selectively block Na^+^ conductance via an interaction with site-1. However, the significant shift in steady-state inactivation suggests a remote allosteric site of action to inhibit ion conductance rather than a pore block. Using a panel of alanine mutants, it was found that the key residues responsible for the interaction of µ-TRTX-Hhn1b (formerly HNTX-IV), a structurally related toxin with similar actions on mammalian Na_V_ channels, are Lys27, Arg29, His28, Lys32, Phe5 and Trp30 [103]. Interestingly, His28 is substituted by the negatively charged Asp26 in µ-TRTX-Hhn2b, thus providing a possible molecular basis for the selectivity of µ-TRTX-Hhn2b for the insect Na channel.

#### 8.1.2. Spider-Venom Peptides Targeting Insect NaV Channel Site-3: Gating Modifiers of Inactivation

Site-3 toxins induce a block or slow Na_V_ channel inactivation and are referred to as gating modifiers of inactivation. The block or slowing of Na_V_ inactivation generally produces an excitatory effect due to the increased activity of Na_V_ channels [98,99]. δ-Ctenitoxin-Pn1a [δ-CNTX-Pn1a; formerly Tx4(6–1)] was isolated from *Phoneutria nigriventer* venom and has significant selectivity towards dipterans (ED_50_ of 36 pmol/g) and blattarians (ED_50_ of 95–477 pmol/g), with no neurotoxic effects in lepidopterans or coleopterans [104]. δ-CNTX-Pn1a specificity was further highlighted by the absence of effects on mammalian Na_V_1.2 and Na_V_1.4 channels [104,105]. δ-CNTX-Pn1a is an excitatory toxin resulting in immediate knockdown, with trembling and uncoordinated movements [104]. It has been definitively established that δ-CNTX-Pn1a interacts with site 3 of the insect Na_V_ channel using competition binding assays where it displaces the site-3 ligand BomIV, an α-like scorpion toxin [105]. 

Insecticidal toxins have also been isolated from the Japanese funnel web spider *Macrothele gigas* [106]. In particular, µ-hexatoxin-Mg1a (µ-HXTX-Mg1a; formerly Magi-2) demonstrates high affinity and selectivity for the insect Na_V_ channel that results in flaccid paralysis of insect larvae. In comparison, the toxin fails to induce any neurotoxic symptoms in mice [106]. The displacement of ^125^I-LqhαIT binding, another classical ligand of insect site-3, from cockroach neurons further implies that an interaction with site 3 is likely. µ-HXTX-Mg1a shares 68% sequence identity with an inactive homolog µ-HXTX-Mg1a (formerly Magi-1). A comparative study of these two toxins theorised that the string of cationic residues Lys16–Lys19 in µ-HXTX-Mg1a may be critical for toxin affinity [106].

#### 8.1.3. Spider-Venom Peptides Targeting Insect NaV Channel Site-4: Gating Modifiers of Activation

Toxins interacting with site 4 typically alter the threshold for action potential generation. Hyperpolarising shifts in the voltage dependence of activation result in the activation of Na_V_ channel at, or near, resting membrane potentials, and result in an excitatory phenotype. In contrast, a depolarising shift in activation threshold results in a depressant phenotype due to the greater depolarisation required to open the channel. Consequently, toxins interacting with neurotoxin receptor site 4 are classed as either depressant or excitatory toxins. 

Four insecticidal peptides, δ-amaurobitoxins (δ-AMATX-Pl1a to -Pl1d; formerly PaluIT toxins), from the venom of *Pireneitega luctuosa* demonstrate high selectivity for insect Na_V_ channels [107]. None of the δ-AMATX-Pl1 toxins demonstrate activity following intracerebroventricular injection into mice [107]. Using native and cloned para/tipE insect Na_V_ channels, δ-AMATX-Pl1 toxins have been shown to slow insect Na_V_ channel inactivation without any significant shifts in the voltage dependence of channel activation. However they fail to modulate the activity of mammalian Na_V_1.2 channels at concentrations up to 10 µM [108]. This action is similar to site-3 neurotoxins. Despite this they have been shown to displace the site-4 excitatory scorpion α-toxin, Bj-xtrIT, but not the site-3 ligand LqhαIT, on cockroach membranes [109]. In reciprocal experiments, Bj-xtrIT and the depressant scorpion α-toxin LqhIT2 also displaced ^125^I-AMATX-Pl1b binding [109]. Thus δ-AMATX-Pl1 toxins represent the first spider toxins that definitively bind to site-4 on insect Na_V_ channels but modulate Na_V_ channel inactivation, an action typically associated with site-3 toxins.

The active site of the δ-AMATX-Pl1 toxins consists of a discontinuous string of residues. A main hot spot of positively charged Arg residues (8, 26, 32 and 34) surrounded by aromatic Tyr residues (22, 30) stands distinct from another aromatic region (Trp12) that is considered critical for activity [109]. Asp19 also appears to play an important role in maintaining toxin activity, however it seems unimportant for target affinity. A similar feature has been observed on the scorpion toxin Bj-xtrIT with Glu15 playing a part in trapping the Na_V_ channel voltage-sensor during channel activation [110]. An action such as this may, in part, account for disparity between toxin activity and target affinity. These results contribute to the theory that the channel target site is more complex than originally perceived.

The μ-agatoxin-Aa1 toxins (µ-AGTX-Aa1a to -Aa1f; formerly µ-Aga I–VI) are a family of terminally amidated 36–38 residue peptides isolated from the venom of the Western grass spider *Agelenopsis aperta* [111,112]. The six members of this family share a high degree of homology with the δ-AMATX-Pl1 toxins [107] and belong to a larger group of µ-agatoxin-1 toxins from *Agelena* *orientalis*, *Agelena opulenta* and *Hololena curta* that are the most potent toxins to modulate the activity of Na_V_ channels [111,112,113,114,115]. µ-Agatoxin-1 family toxins are insect-selective neurotoxins that cause a convulsive paralysis in insects. They are also specific to certain insect orders, being very potent in dipterans (LD_50_ of 30–1380 pmol/g), moderately potent in orthopterans (LD_50_ of 944–4875 pmol/g), but only weakly active in lepidopterans (LD_50_ of 6565–18258 pmol/g). This action is the result of repetitive firing in insect axons resulting in a marked increase in spontaneous neurotransmitter release [111]. This results from a hyperpolarizing shift in the voltage-dependence of Na_V_ channel activation [116,117]. This action is analogous to that reported for site-4 excitatory scorpion β-toxins [118] and therefore it is likely that this family targets site-4, although this awaits further radioligand binding studies. However, µ-AGTX-1 toxins also slow Na_V_ channel inactivation in insect motoneurons [116,117] an action shared by δ-AMATX-Pl1 toxins. The similarities in primary structure and pharmacology of these toxins provide further support for the hypothesis that the insect site-4 is a macrosite, which may be allosterically linked to both channel activation and inactivation. 

#### 8.1.4. Spider-Venom Toxins with an Unknown Site of Action on Insect NaV Channels

A family of 56–59 residue µ-diguetoxin-1 toxins have been isolated from the weaving spider, *Diguetia canities* [119]. These toxins share moderate homology with each other however they do not appear to show any significant homology with any other venom peptides. This family consists of three toxins, isolated as a result of their potent insect paralytic activities, designated µ-DGTX-Dc1a to -Dc1c (formerly DTX9.2, DTX11 and DTX12). µ-DGTX-Dc1a demonstrates strong to moderately potent activity with a PD_50_ value of 380 pmol/g in lepidopterans [119,120]. In mice, μ-DGTX-Dc1a did not show any activity at 657 pmol/g after intraperitoneal injection. While the toxin produced an excitatory effect with increasing muscle spasms until paralysis, the toxin was not lethal. Interestingly, it was apparent that even if larvae recovered from the symptoms of toxicity, feeding was inhibited [119]. This is an important distinction in terms of developing insecticides for crop protection. Studies performed on neuromuscular preparations from *Musca domestica* (house flies) were used to further analyse the rapid and potent activities of these toxins. The application of µ-DGTX-1 toxins induced excitatory postsynaptic potentials [120]. Due to a TTX-dependent effect on cockroach action potentials it is likely that µ-DGTX-1 toxins target insect Na_V_ channels [120], however further studies are necessary to definitively ascertain the site of toxin action. 

### 8.2. Spider-Venom Peptides Targeting Insect Ca_V_ Channels

Ca_V_ channels are key signal transducers that convert depolarization of the cell membrane into an influx of extracellular calcium ions. This ion influx then triggers muscle contraction, hormone and neurotransmitter release, enzymatic activities and patterns of gene expression [99]. Many channel subtypes have been identified in both vertebrates and invertebrates. Insect Ca_V_ channels are divided into two broad families based on their voltage-dependence of activation. Low-voltage-activated (LVA) Ca_V_ channels are activated by small membrane depolarizations and show rapid voltage-dependent inactivation, whereas high-voltage-activated (HVA) Ca_V_ channels are only activated by larger depolarizations and inactivate more slowly.

HVA Ca_V_ channels are composed of a pore forming unit (α_1_), an extracellular subunit (α_2_) linked to a transmembrane δ domain through a disulfide bridge, an intracellular β subunit and a transmembrane γ subunit [121,122]. The transmembrane topology of the α_1_ subunit of Ca_V_ channels is similar to Na_V_ channels and voltage dependence of activation is modulated by a similar mechanism [123]. In contrast, LVA Ca_V_ channels are simpler in structure as they appear to comprise only the α_1_ subunit [121,122].

Insects have a much smaller repertoire of Ca_V_ channels than vertebrates. For example, whereas the human genome encodes 10 pore-forming α_1_ subunits, four β subunits, four α_2_-δ complexes and seven γ subunits, the genome of the fruit fly *Drosophila melanogaster* appears to encode only three α_1_ subunits, a single β subunit, three α_2_-δ subunits and possibly a single γ subunit [124]. However, insects are able to expand their array of functional Ca_V_ channels through alternative splicing and RNA editing [59]. Amino acid sequence comparisons indicate that the three α_1_ subunits produced by *Drosophila*, designated Dmca1D, Dmca1A and Ca-α1T, can likely be classified as HVA Ca_V_1-, and Ca_V_2- channels and LVA Ca_V_3-type channels, respectively [59]. The fact that insects express only a single ortholog of each of three subtypes of Ca_V_ channels [95] might explain why loss-of-function mutations in the genes encoding Dmca1D and Dmca1A are embryonic lethal [125,126]. In contrast, the larger repertoire of Ca_V_ channels in vertebrates permits at least some functional plasticity since mice that harbour a knockout of the gene encoding the α_1_ subunit of many Ca_V_ channel subtypes are viable [95]. This critical role in insects, coupled with <68% homology with their vertebrate counterparts and substantial differences in pharmacological sensitivities [127], makes insect Ca_V_ channels an ideal target for the development of bioinsecticides. However the weaker conservation of insect Ca_V_ channels across insect orders [95] suggests that it might be more challenging to develop blockers of these channels that have a broad spectrum of activity. Of course, the potentially beneficial corollary of this is that it may be easier to develop Ca_V_ channel blockers that target specific groups of insect pests without harming beneficial insects such as pollinators. 

#### 8.2.1. Spider-Venom Peptides that Block Insect Ca_V_1 Channels

ω-Hexatoxin-Hv1a (ω-HXTX-Hv1a, formerly ω-atracotoxin-Hv1a) is the prototypic member of a large family of toxins from the venom of Australian funnel-web spiders with high affinity and specificity for insect Ca_V_ channels [128,129,130,131] (Figure 3Da). These toxins have low ED_50_ values in Orthoptera, Hemiptera, Dictyoptera, Diptera, Coleoptera, Acarina and Lepidoptera [128,129,132] with no effect in vertebrates at up to 10,000-fold higher concentrations [129,130,131,133]. It has been proposed that insect Ca_V_1 channels are the primary target of ω-HXTX-Hv1a [134]. However, recent studies revealed that ω-HXTX-Hv1a is a moderately potent blocker of both MVA and HVA (putative Ca_V_2) currents in cockroach DUM neurons [131]. Thus it appears that ω-HXTX-Hv1a has high affinity for insect Ca_V_1 channels (which may not be present, or present only at very low levels, in DUM neurons) and only moderate affinity for Ca_V_2 channels. Thus, ω-HXTX-Hv1a might be a useful pharmacological agent for simultaneous block of all insect HVA Ca_V_ channel subtypes. In contrast to its effect on insect HVA channels, the toxin has no effect on calcium currents in rat trigeminal neurons [128], nor does it block rat Ca_V_1.2, Ca_V_2.1 and Ca_V_2.2 HVA channels at concentrations as high as 10 μM [135]. Transgenic expression of ω-HXTX-Hv1a in tobacco plants results in protection from *Helicoverpa armigera* and *Spodoptera littoralis* larvae [133]. Topical application of recombinant thioredoxin-ω-HXTX-Hv1a has also been shown to be lethal to these caterpillar species [133]. Of particular interest is the orally active properties demonstrated by ω-HXTX-Hv1a in ticks [136].

#### 8.2.2. Spider-Venom Peptides that Block Insect Ca_V_2 Channels

ω-Plectotoxin-Pt1a (ω-PLTX-Pt1a, formerly Plectreurys toxin-II) is a toxin from the venom of *Plectreurys tristis* containing an unusual *C-*terminal O-palmitoyl threonine amide residue that is critical for toxin activity [137,138,139]. The toxin is assumed to be insecticidal as it blocks presynaptic Ca_V_ channel currents in *Drosophila* nerve terminals [138,140], most likely through specific block of the Ca_V_2 (Dmca1A) channel [141]. This results in a block of neurotransmitter release [138]. In contrast, ω-PLTX-Pt1a has no effect on K_V_ and Na_V_ channels [141] and it fails to block synaptic transmission at frog neuromuscular junctions [140]. At higher concentrations ω-PLTX-Pt1a also begins to disrupt endocytosis [141], suggesting that it might block additional insect Ca_V_ channel subtypes. Nevertheless, low concentrations of ω-PLTX-Pt1a appear to be a defining pharmacology for the *Drosophila*, and possibly other, insect Ca_V_2 channels. 

A second family of insect-selective neurotoxins have also been isolated from *Hadronyche versuta* with a 10,000-fold preference for insect over vertebrate Ca_V_ channels. ω-Hexatoxin-Hv2a (ω-HXTX-Hv2a, formerly ω-atracotoxin-Hv2a) is the prototypic member of a family of 42–45-residue insect-selective neurotoxins [142]. ω-HXTX-Hv2a induces immediate and sustained paralysis when injected into crickets with an ED_50_ of 160 pmol/g [142]. This contrasts with the slow onset of paralysis following injection of ω-HXTX-Hv1a [128]. The toxin is lethal to ticks [136] but it causes no adverse effects when injected into newborn mice [142]. Injection of ω-HXTX-Hv2a into insects induces instantaneous paralysis and application of picomolar doses of toxin results in significant inhibition of Ca_V_ channel currents in bee brain neurons (IC_50_ 130 pM) [142]. ω-HXTX-Hv2a is considered the most potent blocker of insect Ca_V_ channels reported thus far. The insect Ca_V_ channel subtype targeted by ω-HXTX-Hv2a has not been determined, but several lines of evidence suggest it is likely to be Ca_V_2 (reviewed in reference [59]). Unfortunately a recombinant expression system has never been developed for ω-HXTX-Hv2a so this is yet to be confirmed.

ω-Theraphotoxin-Hh2a (ω-TRTX-Hh2a, formerly huwentoxin-V) is a 35-residue peptide toxin isolated from the venom of the Chinese tarantula *Haplopelma schmidti* [143]. Small amounts of toxin induce a reversible paralysis when injected into locusts and cockroaches (PD_50_ = 4 nmol/g) whereas much larger doses (>24 nmol/g) are lethal. The neurotoxic effects of the peptide appear to be insect-specific since mice injected with high doses of toxin (7–49 nmol/g) via the intra-abdominal or intracerebroventricular route are unaffected [143]. ω-TRTX-Hh2a has no effect on Na_V_, K_V_ and MVA Ca_V_ channel currents in cockroach DUM neurons, but it blocks HVA Ca_V_ currents with an IC_50_ of 219 nM [144]. This toxin therefore appears to be a moderately potent, but selective, blocker of insect Ca_V_2 channels, although its effect on insect Ca_V_1 and Ca_V_3 channels remains to be examined. Nevertheless, ω-TRTX-Hh2a might prove to be a valuable pharmacological tool for the study of insect Ca_V_2 channels, especially for dissecting out currents mediated by different Ca_V_2 isoforms.

#### 8.2.3. Spider-Venom Peptides that Block Insect Ca_V_3 Channels

To date, no toxins have been described that block insect Ca_V_3 channels. Furthermore, biophysical and pharmacological characterization of these channels is sadly lacking, with not a single study of insect Ca_V_3 channels reported in the scientific literature. It remains to be determined whether some of the LVA Ca_V_ currents recorded from insect neurons are mediated by Ca_V_3 channels.

### 8.3. Spider-Venom Peptides Targeting Insect K_V_ Channels

Voltage-activated potassium (K_V_) channels are involved in cellular signaling processes, regulation of neurotransmitter release and heart rate, insulin secretion, neuronal excitability, epithelial electrolyte transport, smooth muscle contraction and cell volume regulation [145]. Given this highly diverse range of functions it is not surprising that more than 75 human genes encoding various K_V_ channel subunits have been cloned [146]. There are six known families of voltage-activated potassium (K_V_) channels in *Drosophila*. These have been classified as Shaker (mammalian K_V_1-related), Shaw (K_V_2-related), Shal (K_V_3-related), Shab (K_V_4-related), and EAG, ERG, and ELK (KCNH-related) [147]. Additionally, the *slo* gene family encodes large-conductance, Ca^2+^-activated K_V_ (BK_Ca_) channels and Na^+^-activated K_V_ (K_Na_) channels. These channels activate in response to voltage changes, as well as to changes in the intracellular concentration of calcium (BK_Ca_) and sodium (K_Na_) [148,149]. 

Unlike Na_V_ and Ca_V_ channels, K_V_ channels are tetramers with a four-fold symmetry around a central pore [99]. Each subunit consists of an α-helical transmembrane domain that is made of six transmembrane segments (S1–S6) arranged into two types of domains: a single pore domain formed by the S5–S6 regions from the four subunits, and four surrounding voltage-sensing domains (S1–S4) from a single subunit [99]. The pore domain contains the K^+^-selective ion conduction pathway and the receptor for pore-blocking toxins that bind to the extracellular vestibule near the selectivity filter. BK_Ca_ channels are an exception as they have seven transmembrane segments [149]. K_V_ channels may contain homotetrameric or heterotetrameric α-subunit assemblies, explaining the diversity of these ion channels [150], although Shaker, Shal, Shab and Shaw are all homotetramers [150]. While there are many K_V_ channel families, only one is of importance to this discussion—the insect BK_Ca_ channel.

κ-Hexatoxin-1 family of toxins, isolated from Australian funnel-web spiders [78], were the first spider toxins discovered to selectively target insect potassium channels [151]. These 33–37 residue peptides were originally named “Janus-faced” atracotoxins after the two-faced god Janus from Roman mythology due to the striking asymmetric distribution of hydrophobic and charged residues on opposite surfaces of the molecule [152,153]. The most insecticidal member of this family, κ-HXTX-Hv1c, was recently identified as a high affinity blocker (IC_50_of 2 nM) of insect BK_Ca_ channels with a lack of effect on insect Na_V_, Ca_V_ as well as other subtypes of K_V_ channels [151]. Channel block displayed a lack of voltage-dependence, in contrast with many other spider toxins targeting vertebrate K_V_ channels (for a review see reference [154]). Like the μ-agatoxin-1 family, toxins from the κ-hexatoxin-1 family are specific to certain insect orders, being very potent in dipterans (LD_50_ of 91–319 pmol/g), moderately potent in orthopterans (LD_50_ of 167–1022 pmol/g), but only weakly active in lepidopterans (LD_50_ of 3070–3195 pmol/g). However, the κ-hexatoxin-1 family is not toxic against newborn mice, adult rabbits or isolated preparations of chick biventer cervicis or rat vas deferens [151,152,153]. Therefore κ-HXTX-1 toxins are highly insect-selective.

Although several insect-selective hexatoxins have been isolated from the venom of spiders belonging to the family Hexathelidae, there have been only a limited number of investigations of insecticidal toxins from spiders of the family Theraphosidae [102,155]. This is despite the characterization of a number of toxins from theraphosid (“tarantula”) spiders that target mammalian ASICs, MSCs, K_V_, Na_V_, and Ca_V_ channels. Recently a family of three κ-theraphotoxin-Ec2 toxins (κ-TRTX-Ec2a to -Ec2c) from the venom of the East African tarantula *Eucratoscelus constrictus* were found to block insect BK_Ca_ channels [156] without blocking insect K_V_, Na_V_, or Ca_V_ channels. κ-TRTX-Ec2a induces complete paralysis of orthopterans within 5 min, and death within 15 min at 1100 pmol/g. κ-TRTX-Ec2a and κ-TRTX-Ec2b cause no activity in mice after intracranial injection. Interestingly, κ-TRTX-Ec2c, which shows >80% sequence identity with other members of this toxin family, is not insect specific. It induces strong neurotoxic symptoms including convulsions, tonic paralysis, general ataxia, and respiratory paralysis in mice [156]. κ-TRTX-Ec2c could therefore yield insights into which residues are important for insect specificity. It is noteworthy that although these toxins share their target with κ-HXTX-Hv1c, they possess no obvious sequence homology, implying that they may interact with different sites on the insect BK_Ca_ channel. The κ-hexatoxin-1 and κ-theraphotoxin-1 families will be useful for probing the biological role of BK_Ca_ channels in insects and are potential lead compounds for the development of insect-selective biopesticides.

### 8.4. Membrane-Acting Linear Peptides

To date, several groups of cytolytic peptides with antimicrobial activity have been discovered in araneomorph spider venoms. These have been classified as membrane-acting antimicrobial peptides (MAMPs) with the activity prefix “M” (for a review see reference [67]). These cytolytic peptides are short (<50 residues), highly cationic, amphipathic peptides lacking cysteine residues. While these peptides are highly active against Gram-negative and Gram-positive bacteria, the reported insecticidal effect of MAMPs on insects is negligible with very high LD_50_ values (5–10 nmol/g). In contrast to these short MAMPs, *Lachesana tarabaevi* (Zodariidae) venom also contains a family of long, linear M-ZDTX-Lt toxins (cyto-insectotoxins) with much more potent insecticidal activity, in addition to cytolytic and antimicrobial activity. These are more than twice the length of typical MAMPs and possess very high charge at neutral pH. They still retain an α-helical motif but appear to be composed of two short MAMPs joined together in a “head-to-tail” configuration by a short four-residue linker [157]. 

### 8.5. Spider-Venom Toxins Targeting Presynaptic Nerve Terminals

Venoms from widow spiders of the genus *Latrodectus* (Theridiidae) contain five insect-specific proteins, known as latroinsectotoxins (LIT) α, β, γ, δ and ε [158,159], with phylum-selective insecticidal actions. There is also a vertebrate-specific neurotoxin, α-latrotoxin (α-LTX; for a review see reference [160]), and a toxin affecting crustaceans, α-latrocrustatoxin (α-LCT) [161]. Two of the latroinsectoxins have been cloned and fully sequenced: α-LIT-Lt1a [162] and δ-LIT-Lt1a [163]. They are high-molecular mass proteins with masses of 111 and 130 kDa, respectively. All latrotoxins whose structures have been determined are highly homologous and have a similar domain architecture, which consists of a unique *N-*terminal sequence and a large domain composed of 13–22 ankyrin repeats. It is believed that these toxins induce paralysis in insect prey by stimulating massive neurotransmitter exocytosis from nerve terminals. They act by (i) binding to specific receptors, some of which cause exocytosis, and (ii) inserting themselves into the terminal membrane to form non-selective cation channels (for a review see reference [164]). Specific receptors for LITs have yet to be identified, but all three classes of vertebrate receptors known to bind α-LTX are also present in insects. LITs are the most potent spider-venom toxins known, with LD_50_ values of <1 pmol/g in lepidopterans and dipterans [163,165]. Furthermore, as LITs form ion channels upon membrane insertion it is unlikely that only a short fragment of the protein can be found that mimics the broad range of activity of the entire toxin, given that this fragment would need to encode information regarding targeting, membrane insertion and oligomerization.

### 8.6. Spider-Venom Toxins Targeting Glutamate Receptors

In vertebrates the neuronal actions of L-glutamate are mediated by two distinct glutamate neurotransmitter receptor classes in the CNS: ionotropic [166] and metabotropic [167]. All known vertebrate ionotropic L-glutamate receptors comprise cation channels and are classified into three subtypes: α-amino-3-hydroxyl-5-methyl-4-isoxazole-propionate (AMPA), kainate (KA) and *N*-methyl-D-aspartate (NMDA) receptors. In insects, however, at least two ionotropic L-glutamate-gated cation channels mediate neurotransmission at the neuromuscular junction, rather than in the CNS [168,169]. In addition, two insect ionotropic L-glutamate receptors that gate chloride channels (GluCls) have been discovered in insect CNS neurons [170]. These anion-gated GluCls are important sites of action of insecticides such as ivermectin [171], and in some cases fipronil [170]. 

While there have been a wide range of glutamate receptor antagonists found in spider venoms these are mostly acylpolyamines (in particular α-agatoxins [172]). Thus far, only one spider toxin, Г-CNTX-Pn1a (formerly PnTx4 (5–5)) from the venom of the Brazilian armed spider *Phoneutria nigriventer*, has been found to inhibit vertebrate glutamate receptors, in this case specifically inhibiting NMDA- but not the KA- or AMPA-subtypes [173]. Г-CNTX-Pn1a is highly insecticidal, with <50 pmol/g causing neurotoxic effects immediately after intrathoracic injection in blattarians, orthopterans, and dipterans. This suggests that the effects of Г-CNTX-Pn1a may be mediated via insect glutamate receptors. Importantly, Γ-CNTX-Pn1a had no effect when injected i.c.v. into mice at a dose of 290 pmol/g [173]. 

## 9. Bioinsecticide Lead Selection

There are several requirements for a spider-venom peptide to be considered as a bioinsecticide lead. The most obvious is their potency on the insect target(s). The potency of a toxin is inversely proportional to the volume needed to be deployed in the field. However, potency alone is not sufficient, as selectivity is also crucial. For example, a toxin that is potent on insects but also lethal to vertebrates would not be considered an appropriate bioinsecticide lead, although it might still be used to elucidate the key residues for determining phyletic selectivity. Good examples of such non-phylum-selective toxins are the δ-hexatoxins-1 toxins from Australian funnel-web spiders. δ-Hexatoxin-Ar1a and -Hv1a are potent insecticides, but they are also responsible for the lethal effect of these hexathelid venoms in primates [174]. The most potent insecticidal toxins reported so far are the α- and δ-latroinsectotoxins from *L.* *tredecimguttatus* with LD_50_ values of 0.11 pmol/g and 0.45–0.54 pmol/g, respectively, in lepidopterans. However, with 1170 and 991 residues, respectively, these protein toxins are clearly an exception from the other, mainly low molecular mass insecticidal peptide toxins. Despite their potency and specificity, LITs are not suitable leads for insecticide development as their large size makes it difficult to economically produce the large amounts of protein required for field applications. Among low mass peptide toxins, U_1_-CUTX-As1c from *Apomastus schlingeri* is the most potent in lepidopterans (LD_50_ of 2.4 pmol/g), followed by its paralog U_1_-CUTX-As1d (LD_50_ of 7.2 pmol/g) and U_1_-PLTX-Pt1a from *Plectreurys tristis* with an LD_50_ of 13.8 pmol/g. At the other end of the insecticidal potency scale we find several toxins that target lipid bilayers, with the lowest activity being recorded for M-OXTX-Ot2a from *Oxyopes takobius* with a LD_50_ of 500,400 pmol/g in lepidopterans (*i.e.*, 208,500 times less potent than U_1_-CUTX-As1c). Therefore, it has to be questioned whether such weakly insecticidal peptides should still be considered “insecticidal toxins”.

Ideally, a toxin should also not target all insects but rather only a narrow range of pest species while not harming other arthropods (e.g., pollinators and natural predators of the target pest species). Unfortunately, most studies have failed to determine if insecticidal spider toxins are toxic to beneficial or protected species of certain beetles, dipterans and lepidopterans. Such assays would alleviate environmental concerns over poor insecticidal selectivity. However, the high sequence homology of a single target (e.g., Na_V_ channels) between insect orders suggests that this might be difficult to achieve [95]. Despite this, potency differences have been observed in acute toxicity assays across different insect orders. Based on the phyletic selectivity data in the ArachnoServer 2.0 Database [174], spider toxins have been described with activities against five insect orders: Blattaria, Coleoptera, Diptera, Lepidoptera, and Orthoptera. The LD_50_ values of the same toxin tested in different insect orders can vary by several orders of magnitude. For example, μ-AGTX-Aa1d was reported to be 317 times more potent in dipterans than in lepidopterans. But even within one insect order, activity can vary considerably. For example, U_1_-PLTX-Pt1a is 309 times more potent against *Manduca sexta* than its lepidopteran relative *Heliothis virescens*.

It is tempting to compare literature values for toxin potencies to provide an indication of which toxins might be regarded as potent, medium-strength, or weak insecticides. However, there is neither a standard assay nor standard target species for testing the insecticidal activity of toxins. Factors that can vary include the end-point that is measured (*i.e.*, ED_50_, PD_50_, or LD_50_), the time interval between toxin administration and the end-point, the routes of toxin application, the insect species tested, and different developmental stages/sizes of the insect tested. It is therefore rather difficult to make a direct comparison of the activities of two toxins unless they have been tested in exactly the same experimental setup using the same target species and developmental stage. Nevertheless, the values recorded for insecticidal activity in the ArachnoServer 2.0 Database show a remarkable variation.

There is also a requirement that the toxin is orally- or contact-active, unless the toxin is to be delivered via a vehicle such as an entomopathogen. Unfortunately, the insecticidal activity of most spider-venom peptides has been determined by injection, and their contact and *per os* activity in most cases is unknown (an exception being the ω-HXTX-1 toxins). A further requirement is that the toxin is sufficiently stable under field conditions to affect the target species, while allowing a complete degradation to innocuous metabolites over time to avoid adverse impacts on the environment via biomagnification and bioaccumulation.

Despite the discovery of 136 insecticidal spider toxins, so far less than 25 have been sufficiently characterized (*i.e.*, determination of the full sequence and molecular target) and found to be sufficiently potent and specific for insects to be considered suitable as bioinsecticide leads. It must be noted that some of these toxins have poorly characterised orthologs/paralogs which, given their high sequence homology, may also be suitable bioinsecticide leads. Nevertheless, there are still some promising candidates fulfilling most, or all, of the criteria defined above for an ideal bioinsecticide. Based on the information available to date, we would consider the following spider toxins as suitable candidates for insecticide leads: ω-hexatoxin-1 family, μ-agatoxin-1 family, δ-ctenitoxin-Pn1 family, μ-diguetoxin-Dc1, κ-hexatoxin-1 family, and Γ-CNTX-Pn1a (Table 1). There might be additional candidates among those toxins that are potently insecticidal but for which the molecular target is currently unknown (“U” prefix). More work needs to be done to fully characterize these toxins before their suitability as insecticide leads can be properly gauged. Based only on their insecticidal potency, the most interesting toxin groups for further investigation are (where the number in brackets indicates the total number (*n*) of paralogs/orthologs): (i) the U_1_-, U_2_-, and U_3_-cyrtautoxin-1 family (*n* = 6); (ii) the U_2_-filistatoxin-1 family (*n* = 2); and (iii) the U_1_-plectotoxin-1 family (*n*= 6).

## 10. Commercialisation of Spider-Venom Toxins

The high phyletic specificity, potency, and novel mode of action, of a limited range of spider toxins recommends them as lead compounds for the development of bioinsecticides. Indeed, transgenes encoding insect-specific arachnid toxins, including spider neurotoxins, have been successfully expressed in a number of crops and entomopathogens. One of the simplest ways is via the development of a recombinant baculovirus. Insertion of the gene encoding the toxin into the baculovirus genome greatly enhances the efficacy of natural insect-specific baculoviruses, reducing the “time-to-kill”, and thus increasing the insecticidal potential of these viruses [175,176]. The most widely used baculovirus strain for gene insertion studies has been the *Autographa californica* nuclear polyhedrosis virus (AcNPV) as it infects various important lepidopteran pest insects [177] (for a review see reference [178]). Three spider toxins have already been trialed including μ-AGTX-Aa1d and two toxins whose target has not been clearly defined, U_1_-AGTX-Ta1a (formerly TalTX-1 from the hobo spider *Tegenaria agrestis*) and µ-DGTX-Dc1d [57,175,179,180,181]. Insertion of genes that encode for these toxins has been shown to cause dramatic improvements in the speed of action, causing lepidopteran larvae to die up to 50% more rapidly than those larvae infected with wild-type virus. A similar approach is to engineer a transgene encoding a spider toxin into an entomopathogenic fungus such as *Metarhizium anisopliae* as has been achieved with the insect-selective scorpion toxin AaIT. The toxicity of this fungus was significantly increased agains *t* the tobacco hornworm *Manduca sexta* (Lepidoptera: Sphingidae) and the dengue mosquito *Aedes aegypti* (Diptera: Culicidae) without compromising its host specificity [182]. Although generally regarded as safe and selective, baculoviruses have not met their full potential. Construction of recombinant baculoviruses to speed the time-to-kill pest insects has been validated a number of times, yet they not become a mainstream pest management strategy due to what is often dubbed the “psychological effects of seemingly unsuccessful commercialization” [178].

**Table 1:** Spider peptide toxins suitable as insecticidal leads.

Toxin Name	Source	Insect Target	Acute toxicity test species (Order ^†^: Genus species)	ED_50_ or PD_50_ (pmol/g)	LD_50_ (pmol/g)	Paralogs/orthologs	
δ-CNTX-Pn1a	*Phoneutria nigriventer*	Na_V_ channel	B: *Periplaneta americana*	95 ^‡^		2	
			D: *Musca domestica*	36			
Γ-CNTX-Pn1a	*Phoneutria nigriventer*	GluR	B: *Periplaneta americana*	48 ^‡^		0	
			D: *Musca domestica*	10 ^‡^			
			O: *Acheta domesticus*	29 ^‡^			
κ-HXTX-Hv1c	*Hadronyche versuta*	BK_Ca_ channel	D: *Musca domestica*		91	6	
			D: *Musca domestica*		319 ^#^		
			D: *Lucilia cuprina*		117 ^#^		
			L: *Heliothis virescens*		3195 ^#^		
			L: *Spodoptera frugiperda*		3070 ^#^		
			O: *Acheta domesticus*		167		
			O: *Acheta domesticus*		1022 ^#^		
μ-AGTX-Aa1d	*Agelenopsis aperta*	Na_V_ channel	D: *Musca domestica*		30	11	
			L: *Manduca sexta*		9524		
μ-DGTX-Dc1a	*Diguetia canities*	Na_V_ channel	L: *Heliothis virescens*	380		3	
ω-HXTX-Hv1a	*Hadronyche versuta*	Ca_V_ channel	D: *Musca domestica*	250 ^#^	77	27	
			L: *Heliothis virescens*				
					89		
			O: *Acheta domesticus*				

^†^ B = Blattaria, D = Diptera, L = Lepidoptera, O = Orthoptera; ^‡^ Not ED_50_/PD_50_ values-Neurotoxic effects noted immediately after intrathoracic injection at this concentration; **^#^**Recombinant toxin.

Further transgenic approaches aim to incorporate insecticidal spider toxins into plants for the control of phytophagous pests, as successfully employed for *Bacillus thuringiensis* Cry proteins (for a review see reference [183]). This possibility arises because of the surprising oral toxicity of ω-ACTX-Hv1a recently reported following incorporation into the tobacco plant *Nicotiana tabacum*. Tobacco plants incorporating the spider-toxin transgene had markedly enhanced resistance to *Heliothis armigera* and *Spodoptera littoralis* (Lepidoptera: Noctuidae) [128,133]. Public reticence in some regions may limit commercial deployment of engineered baculoviruses and transgenic plants. Nevertheless, transgenic crops are widely grown in Argentina, Australia, Brazil, Canada, China, India, Pakistan, Paraguay, South Africa, Uruguay, and the USA. These crops can markedly reduce insecticide use and increase crop yield [184]. Human insecticide poisonings have also been reduced by >75% in China since the introduction of GM crops [185]. Despite these benefits, and reductions in risk, some people remain sceptical of the long-term safety and efficacy of GE products.

Other approaches include the development of orally active acaricidal and insecticidal agents. While ω-ACTX-Hv1a has been reported to be toxic by oral administration to the American lone star tick *Amblyomma americanum* [136] no other spider toxins have been reported to possess oral activity even in the modified gut of ticks. Nevertheless, the bioavailability of these peptides may be increased by coupling them to a carrier protein such as snowdrop lectin (*Galanthus nivalis* *agglutinin*, GNA) or garlic lectins to increase the absorption of toxins across the insect midgut [186,187,188]. For example, fusion of the insecticidal spider toxin U_2_-SGTX-Sf1a (SFI1) to GNA significantly increased its oral toxicity to the tomato moth *Laconobia oleracea* [188] as well as the rice brown planthopper *Nilaparvata lugens* and the peach-potato aphid *Myzus persicae* [189]. Surprisingly, a thioredoxin-ω-HXTX-Hv1a fusion protein was found to be insecticidal in Helicoverpa armigera and Spodoptera littoralis caterpillars by topical application [133] (although the fusion protein was applied topically in a solution containing high levels of imidazole, a compound known to have contact insecticidal activity; [190]). These findings open up a variety of approaches for delivery of insecticidal peptides.

A final alternative could be to design conformationally constrained non-peptide mimetics to be used as foliar sprays. Theoretically, using a non-peptide organic scaffold, the peptide residues critical for binding to the target can be grafted onto a backbone structure to produce a peptidomimetic overcoming the bioavailability issues of peptides penetrating the insect cuticle or gut mucosa. The concept has received limited validation following attempts to develop small-molecule drug leads by “cloning” the functional residues of peptide toxins that block vertebrate Ca_V_ or K_V_ channels [191,192]. 

It should also be particularly interesting in the future to examine the interplay between these peptide toxins and conventional chemical insecticides. For example, in tests with neonate *H. virescens*, the scorpion toxin AaIT acted synergistically with cypermethrin [193]. Moreover, a pyrethroid-resistant strain of *H. virescens* was more susceptible than resistant strains to the effects of a recombinant baculovirus that expressed an AaIT transgene [193]. Pyrethroids and AaIT are neurotoxins that act on the same molecular target, namely the Na_V_ channel. Thus, it is interesting that Na_V_ channel mutations that provide resistance to pyrethroids make the channel more susceptible to toxins that bind the channel at different locations than pyrethroids. This suggests that these peptide toxins might be particularly useful for the control of insect populations that have evolved resistance to commercially available chemical insecticides.

## 11. Concluding Remarks

The pesticide market is a multibillion-dollar industry. Agrochemicals dominate the marketplace with >95% of the market share, but their spectrum of activity is often too broad with significant non-target toxicity. Additionally, the restricted range of targets limits their long-term viability in the face of growing insecticide resistance. Since resistance development should be anticipated for any insecticide [194], the development of new insecticides with specificity and effectiveness against target species, together with minimal non-target toxicity and environmental persistence, will continue to be in demand. Spider-venom peptides are a rich source of potential bioinsecticides that can combine the desirable attributes of high potency, novel target activity, structural stability and phyletic selectivity. Moreover, pharmacological characterisation of spider toxins is revealing novel target sites not previous exploited by conventional agrochemicals, thereby validating new insecticide targets for future screening programs. These peptides can be delivered to insect pests via many different routes, including incorporation of transgenes encoding the peptides into entomopathogens or crop plants.

For venom peptides to play a competitive role in the bioinsecticide market they must: (i) have broad pest-species specificity; (ii) have low toxicity in non-target organisms; (iii) remain in the environment long enough to be effective, but not so long as to induce resistance development within pest species; (iv) be cheap to produce; (v) be easy to formulate and deliver; (vi) be publicly perceived as innocuous; and (vii) be readily accessible to both small farmers as well as large agribusinesses (for a review see reference [195]). Compared with existing agrochemicals, some of these latter goals have yet to be fully achieved with spider-venom peptides (see Table 2), although significant technological improvements continue to emerge. Future research will undoubtedly continue to facilitate the realization of these objectives.

**Table 2:** Criteria for development of competitive insecticides.

Goals	OPs ^†^	Carbamates	Pyrethroids	Insect-selective spider toxins
Broad pest-species specificity	+++	+++	+++	+++
Low toxicity in non-target organisms	+	+	++	+++
Remain in the environment long enough to be effective	+++	+++	++	++
Do not persist in environment to induce resistance development	+	+	++	+++
Cheap to produce	+++	+++	++	++
Easy to formulate and deliver	+++	+++	++	+
Publicly perceived as innocuous	+	+	+++	+
Accessible to small farmers and agribusinesses	+	++	+++	+

^†^ Organophosphates.
